# Timed immersion expiration measures in patients with muscular dystrophies

**DOI:** 10.1186/s40945-020-0074-3

**Published:** 2020-02-18

**Authors:** Mariana Callil Voos, Priscila Santos Albuquerque Goya, Bruna Leal de Freitas, Aline Moço Teixeira Pires, Francis Meire Favero, Fátima Aparecida Caromano

**Affiliations:** 1 Physical Therapy, Speech Therapy and Occupational Therapy Department. Faculty of Medicine, University of São Paulo, Rua Cipotânea 51, São Paulo, SP 05360-000 Brazil; 2 Department of Neurology and Neurosurgery, Faculty of Medicine, Federal University of São Paulo, São Paulo, SP Brazil

**Keywords:** Muscular dystrophies, Muscle strength, Spirometry

## Abstract

**Introduction:**

Muscular dystrophies (MD) cause muscle weakness, affecting motor and respiratory functions. Aquatic activities maintain strength and ventilatory function and may require immersion expiration control.

**Objectives:**

(1) To describe the evolution of timed immersion expiration in patients with MD in one-year follow-up. (2) to describe motor and respiratory outcomes in one-year follow-up. (3) to investigate possible relationships between timed immersion expiration and age, motor and respiratory functions.

**Method:**

Fifty-seven patients with MD (12–35 years, Vignos scale 2–8) were evaluated twice, with one-year interval. Immersion expiration control was timed with a chronometer. Motor function was assessed by Motor Function Measure. The respiratory function was evaluated by spirometry. Analysis of variance compared assessments and Pearson tests investigated relationships between variables and age.

**Results:**

Motor and respiratory functions decreased (*p* < 0.001) but timed immersion expiration was maintained. Timed immersion expiration was not correlated to motor and respiratory functions.

**Conclusion:**

As patients maintained timed immersion expiration in the one-year follow-up, aquatic therapy might be a facilitator for people with MD.

## Introduction

Muscular dystrophies (MD) involve a group of disorders characterized by progressive motor and respiratory functions loss [1–3]. Decreased joint mobility and range of motion occur due to muscle weakness, which also impacts on static and dynamic postural balance control [4]. The maintenance of trunk symmetry is important to preserve functional movements and positioning and to prevent deformities [5], because trunk muscles have crucial role in respiratory functions. The respiratory muscles weakness leads to secondary changes such as atelectasis, decreased lung compliance, ineffective cough and repeated infections [6–8]. Patients with MD, mainly Duchenne muscular dystrophy (DMD), develop a restrictive respiratory pattern [8, 9] due to the reduction of the forced vital capacity (FVC), which implicates a risk for respiratory failure [2]. Respiratory function declines at a rate of 6–11% annually in patients with DMD [10–12].

The guidelines about diagnosis and implementation of multidisciplinary care in DMD state that aquatic exercises should be performed. Therapists should consider the clinical conditions of each patient to choose the best strategies. However, there are no precise information concerning the prescription or monitoring of aquatic exercises [1, 2]. In many rehabilitation centers, aquatic physical therapy in recommended for patients with MD [13–15]. Aquatic exercises are beneficial in the management of musculoskeletal, neurologic and cardiopulmonary problems, which are common in patients with MD [15].

Inspiratory muscle weakness is a major component of many chronic diseases, including MD. Hydrostatic pressure leads to changes in respiratory biomechanics, and the respiratory rate increases, due to an increase in respiratory effort. Therefore, immersion may be challenging for these patients [15]. However, immersion may also be used for respiratory training and rehabilitation [15]. The challenge of inspiratory resistance during neck-depth immersion could raise the respiratory muscular strength and endurance [15].

Although aquatic physical therapy has many appealing qualities to provide physical and mental health, information on implementation and effectiveness in patients with MD is very limited. Breathing exercises during aquatic therapy sessions could maintain or even improve respiratory functions in patients with MD by recruiting respiratory muscles [14, 15]. Besides, as observed in other tasks involving lower and upper limbs muscles, timed immersion mouth expiration (TIME) and timed immersion nose expiration (TINE) may be a clinical tool to evaluate strength and respiratory muscles control during immersion.

No previous study investigated the evolution of timed immersion expiration, nor its relationship with age, motor and respiratory functions. The aim of this study was to describe the evolution of timed immersion expiration in patients with MD in one-year follow-up. As a secondary objective, we searched for eventual relationships between motor, respiratory functions and timed immersion expiration.

## Method

This study was approved by the Ethics Committee of Faculty of Medicine of University of São Paulo, process 254/11. All participants or legal guardians gave consent prior to participation in the study. Children and adolescents gave their informed assent. The study was performed in the Physical Therapy Department of Brazilian Association of Muscular Dystrophies and in the Department of Physical Therapy, Speech Therapy and Occupational Therapy of Faculty of Medicine of University of São Paulo.

### Participants

Fifty-seven patients with MD participated. Their leg function was graded as 2–8, according to Vignos scale [4]**.** All participants were diagnosed by molecular analysis. Forty-one of them were diagnosed with DMD (age 12–35 years, median 16.0 ± 6.2, 10 used only nocturnal noninvasive mechanical ventilation, 5 used noninvasive mechanical ventilation continuously). Sixteen patients were diagnosed with limb-girdle muscular dystrophy (LGMD, age 12–35 years, median 28.0 ± 7.2, 6 used only nocturnal noninvasive mechanical ventilation). Both dystrophies affect proximal muscles, but LGMD affects simultaneously pelvic and scapular girdles and DMD affects the pelvic girdle prior to the scapular girdle. In both cases, weakness increases progressively, from proximal to distal regions [1–3]. All participants underwent physical therapy, respiratory therapy, aquatic therapy, occupational therapy, pedagogic, medical and nutritional supervision in the Brazilian Association of Muscular Dystrophies during the study period. Each session lasted for about one hour.

Patients with DMD were treated with steroids, and dosing was prescribed based on the international medical consensus [1]. All participants were fully adapted to the aquatic physical therapy and attended at least six months of aquatic physical therapy sessions (twice a week) prior to the beginning of the present study. All participants were regularly treated with air stacking techniques (twice a week at the institutions and daily, at home, once a day, by caregivers). Patients were treated with airway clearance devices (cough assist machines) whenever necessary [2].

Patients were treated with conventional and aquatic physical therapy sessions twice a week. Conventional physical therapy involved passive stretching exercises, exercises to recruit trunk, lower and upper limbs muscles, assisted (or, if possible, independent) transferring from prone, supine, sitting, kneeling, half-kneeling, standing and dynamic balance in these postures. TheraBands or 0,5 kg ankle or wrist bracelets were used, if possible. Ambulatory patients also performed gait exercises with the assistance of parallel bars, obstacles and could received visual feedback by mirrors [2].

Aquatic physical therapy sessions targeted trunk, upper and lower limbs control. Breathing exercises aimed to improve respiratory muscles. Dynamic exercises, such as throwing and catching a ball were also performed. Stretching and joint mobilization exercises were performed by the therapist, with the use of floaters or resistance devices whenever necessary [14].

Respiratory and cardiac rates and oxygen saturation were monitored during the sessions. The dosing of respiratory and conventional/ aquatic physical therapy exercises was not controlled. Each therapist determined the therapeutic routines of each session, based on the functional aims and outcomes of each patient. All therapists were experienced therapists from Brazilian Association of Muscular Dystrophies and blinded to the aims of the present study.

### Evaluation

Participants were evaluated twice, with one year-interval between assessments. The physical therapists who performed the tests were not informed about the objectives of the present study. All of them had a minimum of two-years’ experience working fulltime with patients with muscular dystrophies.

The TIME and the TINE were demonstrated by the physical therapist prior to evaluation. Times were measured, in seconds, with a Timex® chronometer. Patients were assessed in a 34 °C swimming pool, 1.26 m deep and with a 6.06 m X 4.14 m area. Patients were positioned with the thorax in immersion (water in the neck level), assisted by a physical therapist when needed. TIME was evaluated by asking the patient to inspire the maximum amount of air possible and to release the air only with the mouth, making bubbles. A nose clip was used to assure that the air was being exhalated only by the mouth. TINE was evaluated by asking the patient to inspire the maximum amount of air possible and release the air with the nose in the water, making bubbles, keeping the mouth closed. A pilot study involving aquatic physical therapists from our institution described very high reliability of TIME (Intraclass correlation coefficients: 0.97 intra-raters and 0.95 inter-raters) and TINE (Intraclass correlation coefficients: 0.95 intra-raters and 0.94 inter-raters).

The motor function was evaluated by the Vignos Scale [4] and the Motor Function Measure [13, 14]. The Vignos scale provides ordinal-level data to assess the lower extremity functions from 1 to 10. Higher scores denote more severe patients. Score 1 means that the patient can walk and climb stairs without assistance, while 10 means that the patient is restricted to bed [4]. The Motor Function Measure consists of quantitative assessment of the motor function, in three dimensions: (D1): standing position and transfers, with 13 items; (D2): axial and proximal motor function, with 12 items; (D3): distal motor function, with seven items, six of which refer to upper limbs. The Motor Function Measure is specific for patients with neuromuscular diseases. Scores range from 0 to 3, as follows: 0: the patient is unable to begin the requested task or maintain the initial position; 1: the patient partially accomplishes the item; 2: the patient partially accomplishes the requested movement or accomplishes it completely, but with imperfections; 3: the patient accomplishes the item completely, with controlled movement (normal) [16, 17].

FVC and the peak expiratory flow (PEF) were measured by spirometry, with the patient in sitting and supine positions [10, 18]. FVC is the amount of air that can be maximally and forcibly expelled from the lungs after a maximal inhalation. FVC was evaluated by asking the patient to perform a maximum inspiration, followed by a maximum forced expiration, with no pause between them [10, 18]. A Koko® expirometer was used. The test followed the lips and mouth closing techniques according to the American Thoracic Society norms. Three measures were collected, and the higher measure was considered. PEF was measured during a maximal mouth exhalation to test the expiratory muscles [19, 20].

Two-way analysis of variance (ANOVA) compared groups (DMD and LGMD) and assessments (initial and final evaluations) of timed immersion expiration, motor function and respiratory function. Pearson correlation tests investigated possible relationships between timed immersion expiration, age and motor and respiratory functions of both groups. We considered the significance level alpha < 0.05. Strong correlations were considered if r ≥ 0.70 and moderate correlations if 0.30 < r < 0.70. *Statistica* 13.0 and SPSS *for Windows®* 17.1 were used in all analyses.

## Results

In DMD group (*n* = 41), the median age was 16 years, the median FVC (sitting) was 75%, and MFM median was 51%. In LGMD group (*n* = 16), the median age was 28 years, the median FVC (sitting) was 88%, and MFM median was 64%. Table 1 shows descriptive statistics of both groups (Table 1).

**Table 1:** Descriptive statistics of LGMD and DMD groups. LGMD: limb girdle muscular dystrophy; DMD: Duchenne muscular dystrophy; MFM: Motor Function Measure; TIME: timed immersion mouth expiration; TINE: timed immersion nose expiration; FVC sitting: percentual forced vital capacity on sitting position; FVC supine: percentual forced vital capacity on supine position; PEF sitting: peak expiratory flow on sitting position (mL/min); PEF supine: peak expiratory flow on supine position (L/min)

Variable	Group	Mean	Minimum	Lower quartile	Median	Upper quartile	Maximum	Standard deviation	Standard Error	*P*-value (T test)
Age (years)	LGMD	25.87	12.00	15.00	28.00	33.00	35.00	7.15	1.79	0.053
	DMD	22.13	12.00	16.00	16.00	29.00	35.00	6.21	0.97	
Vignos (score)	LGMD	5.56	1.00	4.00	6.00	7.00	8.00	2.12	0.53	0.539
	DMD	6.37	2.00	7.00	7.00	8.00	8.00	1.89	0.30	
MFM (score)	LGMD	60.81	19.00	51.00	64.00	74.50	90.00	18.56	4.64	0.661
	DMD	52.24	21.00	40.00	51.00	68.00	85.00	17.15	2.68	
TIME (seconds)	LGMD	29.56	11.00	17.50	31.00	38.50	59.00	13.16	3.29	0.713
	DMD	25.10	5.00	17.00	22.00	30.00	60.00	12.34	1.93	
TINE (seconds)	LGMD	20.81	4.00	13.00	20.50	28.50	40.00	9.86	2.47	0.263
	DMD	18.02	5.00	11.00	13.00	20.00	61.00	12.89	2.01	
FVC sitting	LGMD	77.87	20.00	62.00	88.00	97.50	109.00	26.12	6.53	0.706
	DMD	70.95	18.00	48.00	75.00	99.00	114.00	28.79	4.50	
FVC supine	LGMD	73.50	14.00	59.00	82.50	93.00	96.00	23.12	5.78	0.274
	DMD	66.54	14.00	41.00	68.00	94.00	112.00	30.03	4.69	
PEF sitting	LGMD	356.87	130.00	260.00	380.00	405.00	600.00	137.68	34.42	0.084
	DMD	260.00	50.00	210.00	250.00	300.00	540.00	97.62	15.25	
PEF supine	LGMD	296.25	120.00	200.00	350.00	370.00	510.00	117.52	29.38	0.145
	DMD	236.59	40.00	200.00	220.00	270.00	480.00	91.61	14.31	

### Timed immersion expiration outcomes

**Fig. 1:**
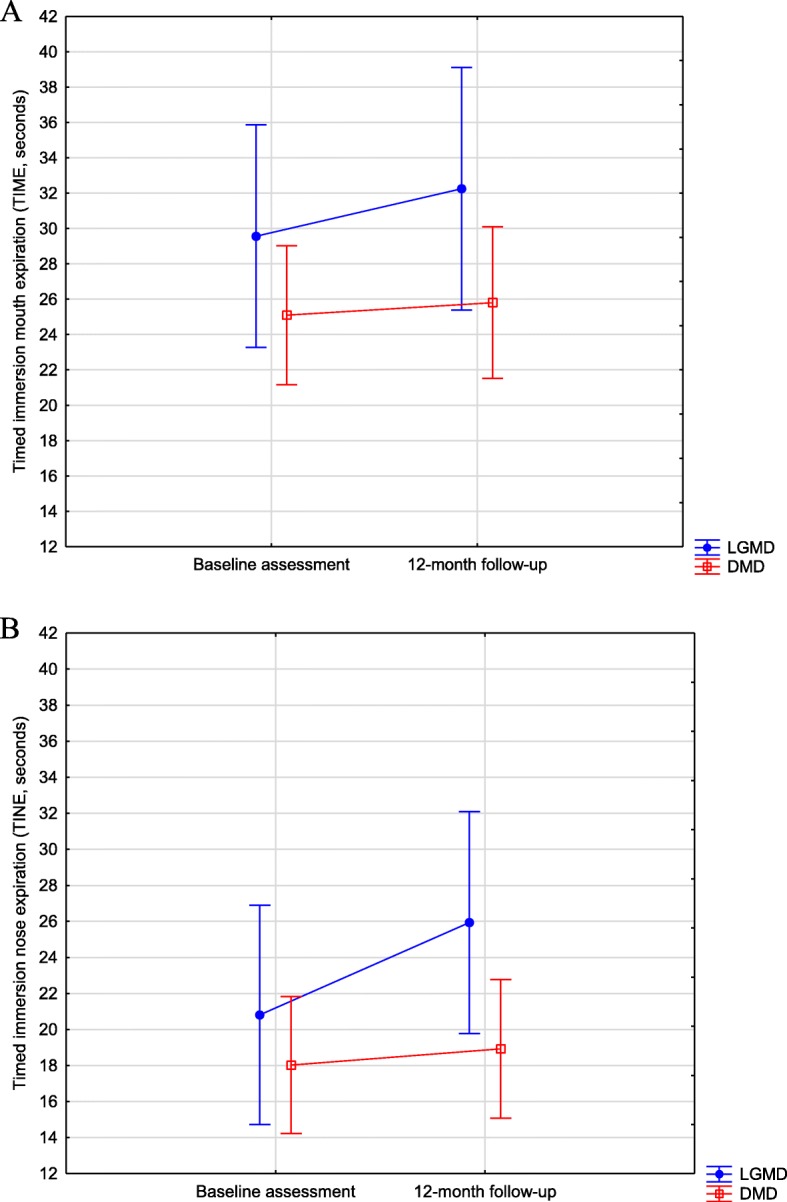
**A** Assessments of timed immersion mouth expiration (in seconds) of limb girdle muscular dystrophy and Duchenne muscular dystrophy groups. **B**: timed immersion nose expiration (in seconds) of limb girdle muscular dystrophy and Duchenne muscular dystrophy groups. Vertical bars denote 95% of the confidence intervals

No significant differences between LGMD and DMD groups were found in TIME and TINE assessments (TIME: F_1,55_ = 2.61; *p* = 0.112 and TINE: F_1,55_ = 2.24; *p* = 0.140). There was no main effect of assessment when baseline assessment and one-year follow-up were compared (F_1,55_ = 0.79; *p* = 0.377) in TIME. However, an effect was observed in TINE, and the one-year follow-up showed significant longer times (F_1,55_ = 4.04; *p* = 0.049). No interactions between groups and assessments were observed (TIME: F_1,55_ = 0.27; *p* = 0.605; TINE: F_1,55_ = 1.98; *p* = 0.165, Fig. 1).

### Motor outcomes

**Fig. 2:**
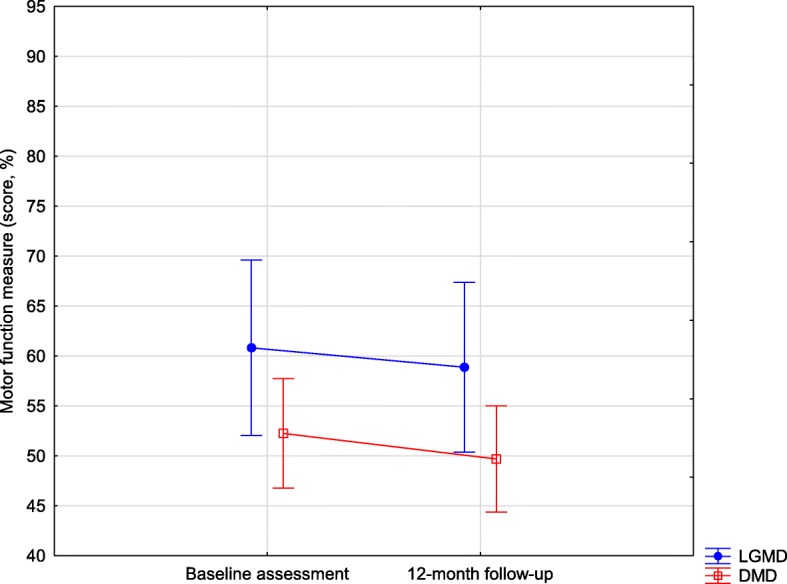
Motor Function Measure (score) of limb girdle muscular dystrophy and Duchenne muscular dystrophy groups. Vertical bars denote 95% of the confidence intervals

No significant difference between LGMD and DMD groups was found in MFM (F_1,55_ = 3.070; *p* = 0.085). Motor function decreased after one year, as the one-year follow-up showed lower scores (F_1,55_ = 20.963; *p* < 0.001). However, there was no interaction between groups and assessments (F_1,55_ = 0.403; *p* = 0.528, Fig. 2).

### Respiratory function measures

**Fig. 3:**
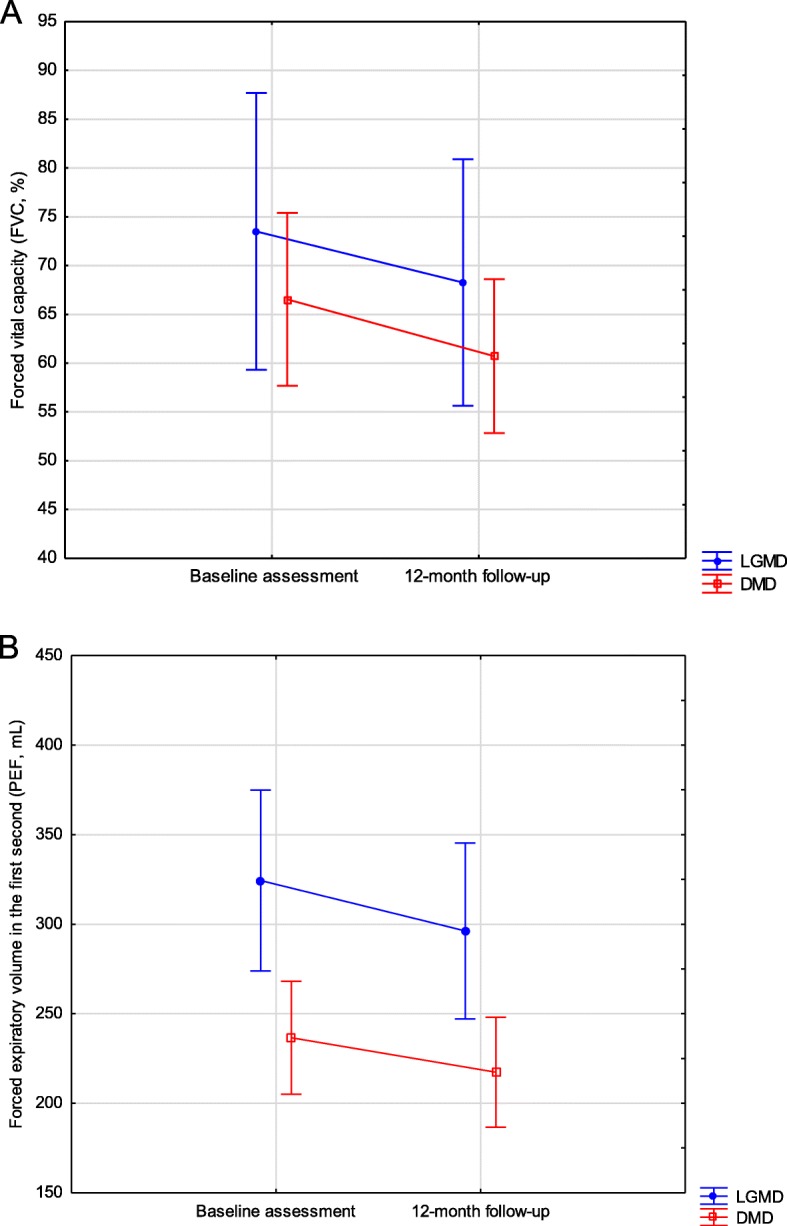
**A**. Forced vital capacity (FVC, %) of limb girdle muscular dystrophy and Duchenne muscular dystrophy groups. **B**. Peak expiratory flow (PEF, mL/min) of limb girdle muscular dystrophy and Duchenne muscular dystrophy groups. Vertical bars denote 95% of the confidence intervals

In FVC assessment, no significant difference between LGMD and DMD groups was found (F_1,55_ = 0.86; *p* = 0.356). However, in PEF assessment, patients with LGMD showed higher measures than patients with DMD (F_1,55_ = 8.16; *p* = 0.006). In both FVC and PEF, significant lower percentages were observed in the one-year follow-up (FVC: F_1,55_ = 19.54; *p* < 0.001; PEF: F_1,55_ = 59.93; *p* < 0.001). No interactions between groups and assessments were observed (FVC: F_1,55_ = 0.05; *p* = 0.818, PEF: F_1,55_ = 2.09; *p* = 0.153, Fig. 3).

In both DMD and LGMD groups, strong correlations between Vignos and MFM, FVC (sitting) and FVC (supine), PEF (sitting) and PEF (supine) were observed. In LGMD group, strong correlations were also observed between MFM and FVC (supine), FVC (supine) and PEF (sitting), FVC (supine) and PEF (supine). In DMD group, TIME showed strong correlation with TINE (Table 2). All Pearson correlation coefficients are displayed in Table 2.

**Table 2:** Pearson correlation coefficients. Relationship between TIME and TINE and motor/respiratory clinical measures (assessment 1). Significance level: alpha< 0.050

DMD		Age	Vignos	MFM	TIME	TINE	FVC sitting	FVC supine	PEF sitting	PEF supine
Age	R		0.184	-0.211	-0.079	0.131	-0.436	-0.460	0.148	0.081
	P		0.267	0.202	0.634	0.433	0.006	0.004	0.373	0.625
Vignos	R	0.184		-0.804	-0.217	-0.303	-0.592	-0.621	-0.322	-0.358
	P	0.267		0.001	0.190	0.064	0.001	0.001	0.048	0.027
MFM	R	-0.211	-0.804		0.231	0.257	0.623	0.621	0.324	0.344
	P	0.202	0.001		0.162	0.119	0.001	0.001	0.047	0.034
TIME	R	-0.079	-0.217	0.231		0.840	0.263	0.269	0.221	0.234
	P	0.634	0.190	0.162		0.001	0.110	0.102	0.181	0.156
TINE	R	0.131	-0.303	0.257	0.840		0.217	0.216	0.247	0.240
	P	0.433	0.064	0.119	0.001		0.190	0.192	0.134	0.145
FVC sitting	R	-0.436	-0.592	0.623	0.263	0.217		0.989	0.634	0.644
	P	0.006	0.001	0.001	0.110	0.190		0.001	0.001	0.001
FVC supine	R	-0.460	-0.621	0.621	0.269	0.216	0.989		0.601	0.616
	P	0.004	0.001	0.001	0.102	0.192	0.001		0.001	0.001
PEF sitting	R	0.148	-0.322	0.324	0.221	0.247	0.634	0.601		0.977
	P	0.373	0.048	0.047	0.181	0.134	0.001	0.001		0.001
PEF supine	R	0.081	-0.358	0.344	0.234	0.240	0.644	0.616	0.977	
	P	0.625	0.027	0.034	0.156	0.145	0.001	0.001	0.001	
LGMD		Age	Vignos	MFM	TIME	TINE	FVC sitting	FVC supine	PEF sitting	PEF supine
Age	R		-0.215	-0.058	0.085	0.129	-0.036	-0.117	0.175	0.176
	P		0.423	0.829	0.753	0.634	0.892	0.665	0.516	0.514
Vignos	R	-0.215		-0.823	-0.238	0.186	-0.580	-0.599	-0.364	-0.320
	P	0.423		0.001	0.375	0.490	0.018	0.014	0.165	0.227
MFM	R	-0.058	-0.823		0.331	-0.127	0.688	0.750	0.499	0.467
	P	0.829	0.001		0.209	0.638	0.003	0.001	0.049	0.068
TIME	R	0.085	-0.238	0.331		0.559	0.383	0.388	0.282	0.224
	P	0.753	0.375	0.209		0.024	0.142	0.137	0.290	0.402
TINE	R	0.129	0.186	-0.127	0.559		0.215	0.204	0.232	0.238
	P	0.634	0.490	0.638	0.024		0.423	0.447	0.387	0.373
FVC sitting	R	-0.036	-0.580	0.688	0.383	0.215		0.933	0.668	0.602
	P	0.892	0.018	0.003	0.142	0.423		0.001	0.005	0.013
FVC supine	R	-0.117	-0.599	0.750	0.388	0.204	0.933		0.743	0.717
	P	0.665	0.014	0.001	0.137	0.447	0.001		0.001	0.002
PEF sitting	R	0.175	-0.364	0.499	0.282	0.232	0.668	0.743		0.971
	P	0.516	0.165	0.049	0.290	0.387	0.005	0.001		0.001
PEF supine	R	0.176	-0.320	0.467	0.224	0.238	0.602	0.717	0.971	
	P	0.514	0.227	0.068	0.402	0.373	0.013	0.002	0.001	

Legend: *MFM* Motor Function Measure; *TIME* timed immersion mouth expiration; *TINE* timed immersion nose expiration; *FVC* forced vital capacity; *PEF* peak expiratory flow; *DMD* Duchenne muscular dystrophy; *LGMD* limb-girdle muscular dystrophy

## Discussion

The present study compared the progression of timed immersion mouth and nose expiration and motor and respiratory functions in patients with MD in one-year follow-up. We also investigated possible relationships between timed immersion mouth and nose expiration and age, motor and respiratory functions. Motor and respiratory functions deteriorated in MD patients in one-year follow-up, as observed in previous studies [21, 22]. However, timed immersion expiration did not deteriorate. To our knowledge this is the first study that describes activities involving respiratory control in immersion in patients with MD. Health professionals usually consider data from the respiratory assessment [23] for clinical decision-making about aquatic therapy for patients with MD. Therefore, the impairment of respiratory function should not limit the practice of aquatic exercises.

Motor and respiratory functions were poorer after one year in patients with MD. However, TIME performance was maintained and TINE performance improved. TIME and TINE correlated to each other, but not to FVC and PEF. Therefore, the expiration control in immersion may diverge from traditional spirometry measures, which are the gold-standard for respiratory function in MD [7–10]. Some patients may show respiratory function impairment in spirometry and good mouth and nose expiratory times in immersion, showing that several compensatory strategies are available in the aquatic environment.

In TIME and TINE assessment, the hydrostatic pressure helps the trunk stability by abdominal muscles, because the thorax is in immersion. Therefore, the diaphragm becomes more efficient and this may explain the better respiratory control at the swimming pool15. Aquatic therapy may be a complement for respiratory and physical therapies. TIME and TINE recruit face and trunk muscles, which can optimize respiratory functions [24] (e.g. glossopharyngeal breathing), eating and swallowing in patients with MD [25, 26].

Recent research protocols in the aquatic environment aim at optimizing trunk control, due to the hydrostatic pressure, which also improves body perception and even proprioception [27]. Aquatic therapy can be beneficial for strength, endurance and range of motion maintenance. Besides, the aquatic environment provides higher social participation, which optimizes emotional control and self-esteem and promotes mental health [14]. Concisely, aquatic therapy can be a facilitator for people with MD. The water can help respiratory muscles to have a better performance than in traditional respiratory therapy on sitting or supine position.

## Conclusion

Motor and respiratory functions decreased in one year in patients with MD. However, timed immersion mouth and nose expiration were maintained or even improved in the same period. Our findings show that aquatic therapy could be safe and feasible for patients with MD, and could also lead to improvement in specific tasks, such as timed immersion expiration.

## Data Availability

The databank generated in this study is available for further analysing and checking if necessary.
